# Prevalence and determinants of virological failure, genetic diversity and drug resistance among people living with HIV in a minority area in China: a population-based study

**DOI:** 10.1186/s12879-020-05124-1

**Published:** 2020-06-23

**Authors:** Dan Yuan, Meijing Liu, Peng Jia, Yiping Li, Yuling Huang, Li Ye, Laze Api, Maogang Chen, Liang Yao, Zixin Wang, Honglu Liu, Shu Liang, Shujuan Yang

**Affiliations:** 1 Center for AIDS/STD Control and Prevention, Sichuan Center for Disease Control and Prevention, Chengdu, China; 2 West China School of Public Health and West China Fourth Hospital, Sichuan University, Chengdu, Sichuan China; 3 Department of Land Surveying and Geo-Informatics, The Hong Kong Polytechnic University, Hong Kong, China; 4 International Initiative on Spatial Lifecourse Epidemiology (ISLE), Hong Kong, China; 5 Butuo Center for Disease Control and Prevention, Butuo, China; 6 Liangshan Center for Disease Control and Prevention, Xichang, China; 7 Butuo County People’s Hospital, Butuo, China; 8 Centre for Health Behaviours Research, The Jockey Club School of Public Health and Primary Care, The Chinese University of Hong Kong, Hong Kong, China

**Keywords:** HIV, Antiretroviral therapy, Virological failure, HIV-1 subtypes, Drug resistance

## Abstract

**Background:**

Liangshan Yi Autonomous Prefecture is one of the areas that most severely affected by human immunodeficiency virus (HIV) in China, and virological failure on antiretroviral therapy (ART) is serious in this area. Analyses of prevalence and determinants of ART failure, the genetic diversity and drug resistance among people living with HIV (PLWH) helps improve HIV treatment efficiency and prevent HIV transmission.

**Methods:**

A total of 5157 PLWH were recruited from 2016 to 2017. The venous blood samples were subjected to RT-PCR, followed by sequencing of the HIV-1 pol gene, targeting the protease and reverse transcriptase fragments. HIV-1 diversity was analyzed using the DNAStar software and drug resistance mutations were analyzed using the Stanford University HIV Drug Resistance Database.

**Results:**

A total of 2156 (41.81%) PLWH showed virological failure on ART. Males (ORm = 1.25), heterosexual behaviors and drug injection (ORm = 1.44) and mother to child transmission routes (ORm = 1.58), the clinical stage of AIDS (ORm = 1.35), having used illicit drugs and shared the needles (1–4 times: ORm = 1.34; more than 5 times: ORm = 1.52), having ever replaced ART regimen (ORm = 1.48) increased the risk of virological failure among PLWH, while higher education lever (ORm = 0.77) and ≥ 12 months on ART (12 ~ 36 months: ORm = 0.72; ≥36 months: ORm = 0.66) was associated with lower likelihood of virological failure. The data revealed that CRF07_BC (1508, 95.62%) were the most common strains, and the drug-resistant rate was 32.10% among PLWH with virological failure in this area. The high frequencies of drug resistance were found in EFV and NVP of NNRTIs, ABC, FTC and 3TC of NRTIs, and TPV/r in PIs. The most common mutations in NNRTIs, NRTIs and PIs were K103N/KN (64.69%), M184V/MV/I (36.29%) and Q58E/QE (4.93%), respectively.

**Conclusion:**

We concluded that surveillance of virological failure, HIV-1 subtypes, and drug resistance to understand HIV-1 epidemiology and guide modification of ART guidelines, and target prevention and control strategies should be formatted to reduce the virological failure and drug resistance to promote viral suppression and prevent HIV-1 transmission.

## Background

Human immunodeficiency virus (HIV) infections in humans affect more than 37 million individuals worldwide, and approximately 0.85 million people living with HIV (PLWH) live in China. The number of PLWH has increased by 14% from 2016 to 2017 [1]. Liangshan Yi Autonomous Prefecture, as the largest traditional settlement of Yi minority people in China, is the area affected most severely by HIV in China [2]. By the end of 2018, there have been 38,545 PLWH reported in Liangshan, and prevalence rates of HIV in five counties were the highest in China [3, 4]. Effective HIV interventions should be formatted to reduce HIV transmission in this area.

Antiretroviral therapy (ART) is highly effective in suppressing HIV, stopping the progression of HIV disease and reducing the risk of HIV transmissions [5, 6]. The rapid scale-up of ART could lead to a significant decline in morbidity and mortality among PLWH, which has been confirmed by many studies [5, 7, 8]. ART is a long-term treatment with the potential for drug toxicity and the emergence of HIV resistance, setting the stage for a suboptimal response or lack of sustained response to therapy that is defined as virological failure. Unfortunately, virological failure not only compromises therapeutic effects for individuals but also endangers the population as a whole [9]. In Liangshan, the virological failure rate is reported to be about 36–45% [10, 11], which is more serious than average in China [12], mainly due to the poor adherence to ART and inadequate drug levels. The high prevalence of virological failure may lead to a large extent of genotypic HIV-1 drug resistance. When an individual is infected with drug resistance mutations, the resistant strain may become the dominant strain for further transmission of HIV-1 drug resistance [13], and consequently increasing the number of antiretroviral-naive patients and reducing their therapeutic effectiveness [14]. Understanding the risk factors of virological failure could help to target HIV prevention and intervention strategies [15].

HIV is characterized by its great genetic variability and responsibility for developing drug-resistant mutations with high viral replication rates. This not only increases the risk of virological failure on antiretroviral treatment, but also results in the spread of drug-resistant strains, which brings a huge challenge to controlling the HIV epidemic and preventing the HIV spread. The genotype of HIV-1 is related to the route of transmission, and there are differences in epidemic scale and distribution characteristics [16, 17]. The HIV-1 genotype distribution had been described in parts of China [18, 19]. However, little data is known about that in the highest HIV epidemic area of China. Therefore, we aimed to analyze prevalence and determinants of ART failure, and understand the genetic diversity and drug resistance among PLWH, which could help us better understand HIV epidemiology and allow a timely modification of ART guidelines in this area.

## Methods

### Study participants

Participants were recruited through a multi-stage cluster sampling strategy. The county with the most severe HIV epidemic was selected from all 17 counties in Liangshan Prefecture. According to the basic information system for Acquired Immune Deficiency Syndrome (AIDS) prevention and treatment, all PLWH in this county were included in our study. We have tried to identify as many PLWH as possible through all three possible channels: (1) Provider-Initiated HIV Testing & Counseling (PITC) service for HIV, (2) Voluntary Counseling and Testing (VCT) service through fixed VCT sites and non-government organizations (NGOs), (3) HIV sentinel surveillance (HSS) for unmarried youths, pregnant women, community population, and drug users. The inclusion criteria for PLWH were: (1) receiving confirmatory HIV diagnosis, (2) being permanent residents or had stayed in Liangshan area for more than 5 years, (3) receiving ART in the local township health center for more than 6 months. The exclusion criteria for participants were having have major psychiatric illness (e.g., schizophrenia and bipolar disorder.

According to the inclusion criteria and exclusion criteria, medical staff phoned candidate participants about the study and confirmed their eligibility to join the study. All participants signed informed consent forms before enrollment. Finally, a total of 5157 PLWH were included into the study.

### Data collection

Questionnaire was used to ask all participants in private rooms to collected the socio-demographic information, possible route of HIV transmission, HIV-related behaviors and disease-related characteristics. The socio-demographic information including gender, age, occupation, current marital status, ethnicity, education level. The route of HIV transmission included drug injection, heterosexual behaviors and drug injection, casual sexual behavior and risk behaviors related to mother-to-child transmission treatment time. The HIV-related behaviors included information about needle sharing, number of casual sexual partners and history of sexually transmitted disease except for HIV. The disease related characteristics were extracted from their medical record, including viral load, stage of the disease, the initial regimen and time of ART and change of ART regimen. The above information was collected from National AIDS reporting system. When a newly diagnosed with HIV-1 infection was found or received ART, the detailed information (the initial regimen and time of ART) would be collected and inputted into system.

### Laboratory tests

Each participant was asked to provide 5 mL of venous blood for detecting the viral load of HIV-1. The plasma samples were isolated from each participant, and preserved in a − 80 °C freezer until analysis. HIV viral load was measured in the Sichuan Center for Disease Control and Prevention. Virological failure in ART was defined as HIV RNA level ≥ 1000 copies/ml [20, 21] after receiving ART for more than 6 months.

### Nucleic acid extraction, amplification, sequencing and drug-resistance analyses

Total viral nucleic acid was extracted from 200-μl plasma using an automatic extraction machine (MagNA Pure LC 2.0 system, Roche, Branchburg, NJ, USA). The full-length protease gene in *Pol* region and the first 300 codons of the reverse transcriptase gene was amplified by using reverse transcription polymerase chain reaction (RT-PCR). The amplified products were purified in and sequenced at Beijing Genomics Research Center Ltd., in China.

The HIV-1 pol sequences obtained in the study, together with reference sequences of different subtypes and CRFs, were edited and aligned using ChromasProl.33, and the sequence alignments were manually performed by BIOEDIT Sequence Alignment Editor software (Ibis Biosciences, Carlsbad, CA, USA). The detailed amplification, sequencing and drug resistance analyses were detailed described previously [22, 23].

### Statistical analysis

Data were analyzed using SPSS version 21.0 for Windows (SPSS, Inc., Chicago, IL, USA). Categorical variables were expressed as frequencies and percentages and compared using the chi-squared (χ^2^) test. Using the presence of virological failure in ART as the dependent variable, univariate odds ratios (ORu) and 95% confidence interval (CI) for socio-demographic information, possible route of HIV transmission, HIV-related behaviors and disease-related characteristics were estimated. A summary model was obtained by fitting a multiple regression model that considered all variables that were significant in the respective univariate analysis as candidates. Multivariate odds ratios (ORm) and respective 95%CI were obtained. Was used for data analysis, with *p* < 0.05 taken as statistically significant.

## Results

### Prevalence and determinants of virological failure on ART among PLWH

A total of 2156 (41.81%) PLWH showed virological failure on ART from 2016 to 2017. Among PLWH with virological failure, majority of them were male, peasant, married, illiteracy, Yi minority, having HIV transmission route of drug injection, the first CD4+ counts of 200 ~ 500 counts/ul, having never used illicit drugs and shared the needle, having no casual sexual partners and history of sexually transmitted diseases except for HIV (Table 1).

**Table 1:** Prevalence and determinants of virological failure on ART among PLWH

Variables	VL ≥ 1000 copies/ml	Total N	ORu	ORm
	Virologic failure *n* = 2156 (rate, n/N%)			
***Socio-demographics***				
**Gender**				
Female	710 (36.90)	1925	1.0(ref)	1.0(ref)
Male	1446 (44.70)	3232	1.39 (1.23–1.56)***	1.25 (1.03–1.50)***
**Age**				
≤ 15	172 (48.86)	352	1.0 (Ref.)	
15 ~ 40	1423 (41.45)	3433	0.74 (0.59–0.92) ***	
>40	533 (40.94)	1302	0.72 (0.57–0.92) ***	–
Unknown	28 (40.00)	70	–	
**Occupation**				
Employed	37 (34.91)	106	1.0 (Ref.)	
Peasant	1736 (41.85)	4148	1.34 (0.90–2.01)	
Students and children	136 (46.74)	291	1.64 (1.03–2.60)**	–
Unknown	247 (40.36)	612	–	
**Current marital status**				
Married	1406 (41.80)	3366	1.0 (Ref.)	
Single	428 (45.77)	934	1.18 (1.02–1.36) **	
Widowed or Divorced	99 (35.87)	276	0.78 (0.60–1.01)*	–
Unknown	223 (38.38)	581	–	
**Ethnicity**	(0.00)			
Yi	2137 (41.70)	5125	1.0 (Ref.)	
Han and others	19 (59.38)	32	2.04 (1.01–4.15)**	–
**Education level**				
Illiteracy	1339 (42.58)	3145	1.0 (Ref.)	1.0 (Ref.)
Primary school	581 (40.77)	1425	0.93 (0.82–1.05)*	0.77 (0.66–0.89)**
Secondary or above	70 (38.67)	181	0.85 (0.63–1.16)	0.69 (0.46–1.04)
Unknown	166 (40.89)	406	–	
**HIV transmission route**				
Heterosexual behaviors	715 (37.10)	1927	1.0 (Ref.)	1.0 (Ref.)
Drug injection	747 (43.08)	1734	1.28 (1.12–1.47)***	1.12 (0.93–1.35)
Heterosexual behaviors and drug injection	325 (49.02)	663	1.63 (1.36–1.95) ***	1.44 (1.15–1.82)**
Mother to child	163 (48.95)	333	1.63 (1.29–2.05) ***	1.58 (1.21–2.08)**
Others and unknown	206 (41.20)	500	–	–
**The first CD4**^**+**^**counts, counts/ul**				
≤ 200	346 (46.82)	739	1.0 (Ref.)	
200 ~ 500	986 (41.29)	2388	0.80 (0.68–0.94)**	
>500	646 (40.68)	1588	0.78 (0.65–0.93)**	–
Unknown	178 (40.27)	442	–	
**Stage of disease**				
HIV	784 (37.44)	2094	1.0 (Ref.)	1.0 (Ref.)
AIDS	985 (44.49)	2214	1.34 (1.19–1.51) ***	1.35 (1.17–1.55)***
Unknown	387 (45.58)	849	–	
***HIV-related behaviors***				
**Needle sharing**				
Had never used illicit drugs and shared the needle	1001 (39.03)	2565	1.0 (Ref.)	1.0 (Ref.)
Had shared needle for 1 ~ 4 times	519 (43.43)	1195	1.20 (1.04–1.38)**	1.34 (1.09–1.65)**
Had shared needle ≥5 times	421 (48.00)	877	1.44 (1.24–1.68) ***	1.52 (1.21–1.91)***
Unknown	215 (41.35)	520	–	
**Number of casual sexual partners**				
0	1089 (42.51)	2562	1.0 (Ref.)	
1 ~ 4	508 (38.99)	1303	0.86 (0.75–0.99)**	
≥ 5	344 (44.56)	772	1.09 (0.92–1.28)	–
Unknown	215 (41.35)	520	–	
**History of sexually transmitted diseases except for HIV**				
Never	1494 (42.53)	3513	1.0 (Ref.)	
Ever	100 (38.46)	260	0.85 (0.65–1.09)	–
***ART related information***				
**Time of ART**				
< 12	304 (43.55)	698	1.0 (Ref.)	1.0 (Ref.)
12 ~ 36	682 (40.38)	1689	0.88 (0.73–1.05)*	0.72 (0.60–0.92)**
> 36	728 (40.00)	1820	0.86 (0.72–1.03)*	0.66 (0.53–0.83)***
Unknown	442 (46.53)	950	–	
**Initial regimen of ART**				
TDF + 3TC + EFV/NVP	1202 (39.97)	3007	1.0 (Ref.)	
AZT + 3TC + EFV/NVP	444 (44.36)	1001	1.20 (1.04–1.38)**	
LPV/r + 3TC + AZT/TDF	48 (33.33)	144	0.75 (0.53–1.07)*	
Unknown	462 (45.97)	1005	–	=
**Replacement of ART regimen**				
Never	1535 (40.16)	3822	1.0 (Ref.)	1.0 (Ref.)
Ever	181 (46.65)	388	1.30 (1.06–1.61)**	1.48 (1.12–1.96)**

*ORu* univariate odds ratios, *ORm* multivariate odds ratios, *CI* confidence interval, *TDF* tenofovir, *3TC* lamivudine, *NVP* nevirapine, *EFV* efavirenz, *AZT* Zidovudine, *LPV/r* fosamprenavir/ritonavir

Variables with *p* < 0.2 in the univariate analysis as candidates were selected by a summary multiple logistic regression model

*P value< 0.2; **P value< 0.05; ***P value< 0.001

In univariate analysis, gender, age, occupation, current marital status, ethnicity, HIV transmission route, CD4^+^ level, stage of disease, needle sharing and the number of casual sexual partners, the initial regimen of ART and change of ART regimen were significantly associated with risk of virological failure on ART (*p* < 0.05). In multivariate logistic regression model, we found that males, heterosexual behaviors and drug injection and mother to child transmission routes, stage of AIDS, having used illicit drugs and shared the needles, having ever replaced ART regimen significantly increased the risk of virological failure among PLWH (*p* < 0.05), while higher education level and time on ART ≥12 months were associated with lower likelihood of virological failure (*p* < 0.05) (Table 1).

### Prevalence of drug resistance among PLWH with virological failure on ART

**Fig. 1:**
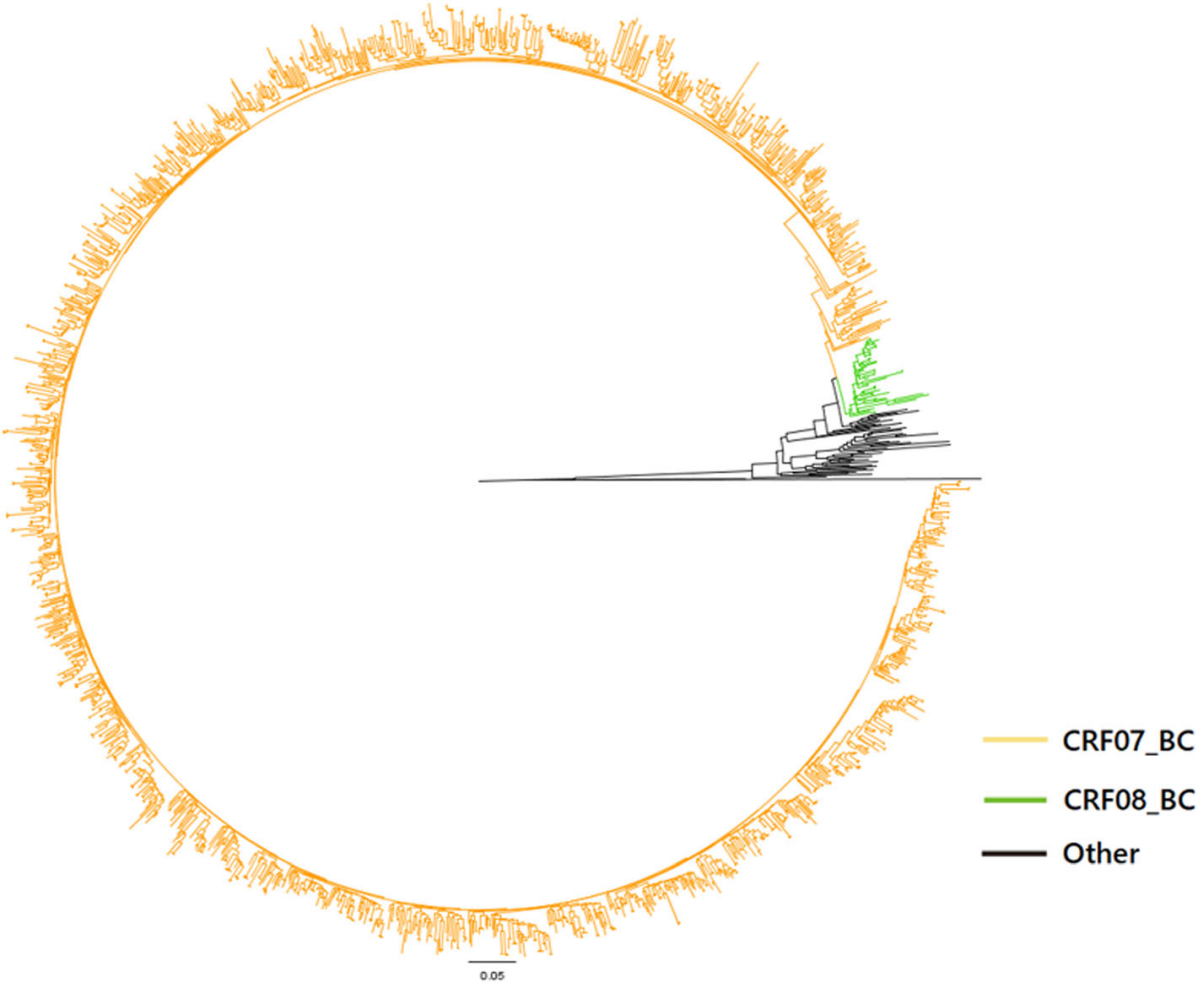
Phylogenetic tree analyses of the HIV-1 pol sequences in PLWH with virological failure on ART; CRF07_BC accounted for 95.62% of the HIV-1 pol sequences; CRF08_BC accounted for 2.66% of the HIV-1 pol sequences; Others were C subtypes and reference strains

Drug-resistant mutation in 1576 (73.10%, 1576/2156) samples were successfully detected. The phylogenetic analyses based on the pol regions showed that CRF07_BC (1508, 95.62%) were the most common strains in this area, and followed by CRF08_BC (42, 2.66%) and C (26, 1.72%) subtypes were the most common strains in Sichuan province (Fig. 1).

Among PLWH with virological failure, the drug-resistant rate was as high as 32.10% (507/1576). The prevalence of drug resistance was high in nonnucleoside reverse transcriptase inhibitors (NNRTIs) in all treatment regimens, then followed by nucleoside reverse transcriptase inhibitors (NRTIs) and protease inhibitors (PIs) (Table 2). The high frequencies of drug resistance were found in Efavirenz (EFV) and Nevirapine (NVP) of NNRTIs, Abacavir (ABC), Emtricitabine (FTC) and Lamivudine (3TC) of NRTIs, and tipranavir / ritonavir (TPV/r) in PIs. The LPV/r + 3TC + AZT/TDF regimen showed the lowest drug resistance to PIs, NRTIs, and NNRTIs compared to the other two regimens.

**Table 2:** Prevalence of drug resistance among PLWH with virological failure on ART

	TDF + 3TC + NVP/EFV	AZT + 3TC + NVP/EFV	LPV/r + 3TC + AZT/TDF	Others	Total
	*n* = 928 (%)	*n* = 318 (%)	*n* = 47 (%)	*N* = 283 (%)	*N* = 1576 (%)
**PIs**	**13 (1.40)**	**9 (2.83)**	**1 (2.13)**	**6 (2.12)**	**35 (2.22)**
ATV/r	–	–	–	1 (0.35)	2 (0.13)
DRV/r	–	–	–	–	–
FPV/r	–	1 (0.31)	–	1 (0.35)	3 (0.19)
IDV/r	–	–	–	1 (0.35)	2 (0.13)
LPV/r	–	–	–	1 (0.35)	2 (0.13)
NFV	1 (0.11)	1 (0.31)	–	2 (0.71)	6 (0.38)
SQV/r	–	–	–	1 (0.35)	2 (0.13)
TPV/r	12 (1.29)	8 (2.52)	1 (2.13)	5 (1.77)	31 (1.97)
**NRTIs**	**97 (10.45)**	**55 (17.30)**	**2 (4.26)**	**39 (13.78)**	**232 (14.72)**
ABC	92 (9.91)	55 (17.30)	2 (4.26)	39 (13.78)	227 (14.40)
AZT	26 (2.80)	22 (6.92)	2 (4.26)	8 (2.83)	66 (4.19)
D4T	44 (4.74)	26 (8.18)	2 (4.26)	19 (6.71)	110 (6.98)
DDI	45 (4.85)	25 (7.86)	2 (4.26)	19 (6.71)	110 (6.98)
FTC	91 (9.81)	55 (17.30)	2 (4.26)	39 (13.78)	226 (14.34)
3TC	91 (9.81)	55 (17.30)	2 (4.26)	39 (13.78)	226 (14.34)
TDF	30 (3.23)	18 (5.66)	1 (2.13)	16 (5.65)	81 (5.14)
**NNRTIs**	**269 (28.99)**	**108 (33.96)**	**10 (21.28)**	**90 (31.80)**	**567 (35.98)**
EFV	261 (28.13)	103 (32.39)	10 (21.28)	88 (31.10)	550 (34.90)
ETR	63 (6.79)	42 (13.21)	1 (2.13)	24 (8.48)	154 (9.77)
NVP	261 (28.13)	105 (33.02)	10 (21.28)	88 (31.10)	552 (35.03)
RPV	96 (10.34)	58 (18.24)	1 (2.13)	35 (12.37)	225 (14.28)

*TDF* Tenofovir, *3TC* Lamivudine, *NVP* Nevirapine, *EFV* Efavirenz, *AZT* Zidovudine, *LPV/r* Fosamprenavir/ritonavir, *ATV/r* Atazanavir/ritonavir, *DRv/r* Darunavir/ritonavir, *FPV/r* Fosamprenavir/ritonavir, *IDV/r* Indinavir/Ritonavir, *NFV* Nelfinavir, *SQV/r* Ritonavir-boosted saquinavir, *TPV/r* Tipranavir/ritonavir, *ABC* Abacavir, *D4T* Stavudine, *DDI* Didanosine, *FTC* Emtricitabine, *ETR* Etravirine, *RPV* Rilpivirine

### Antiretrovairal resistance mutations

**Fig. 2:**
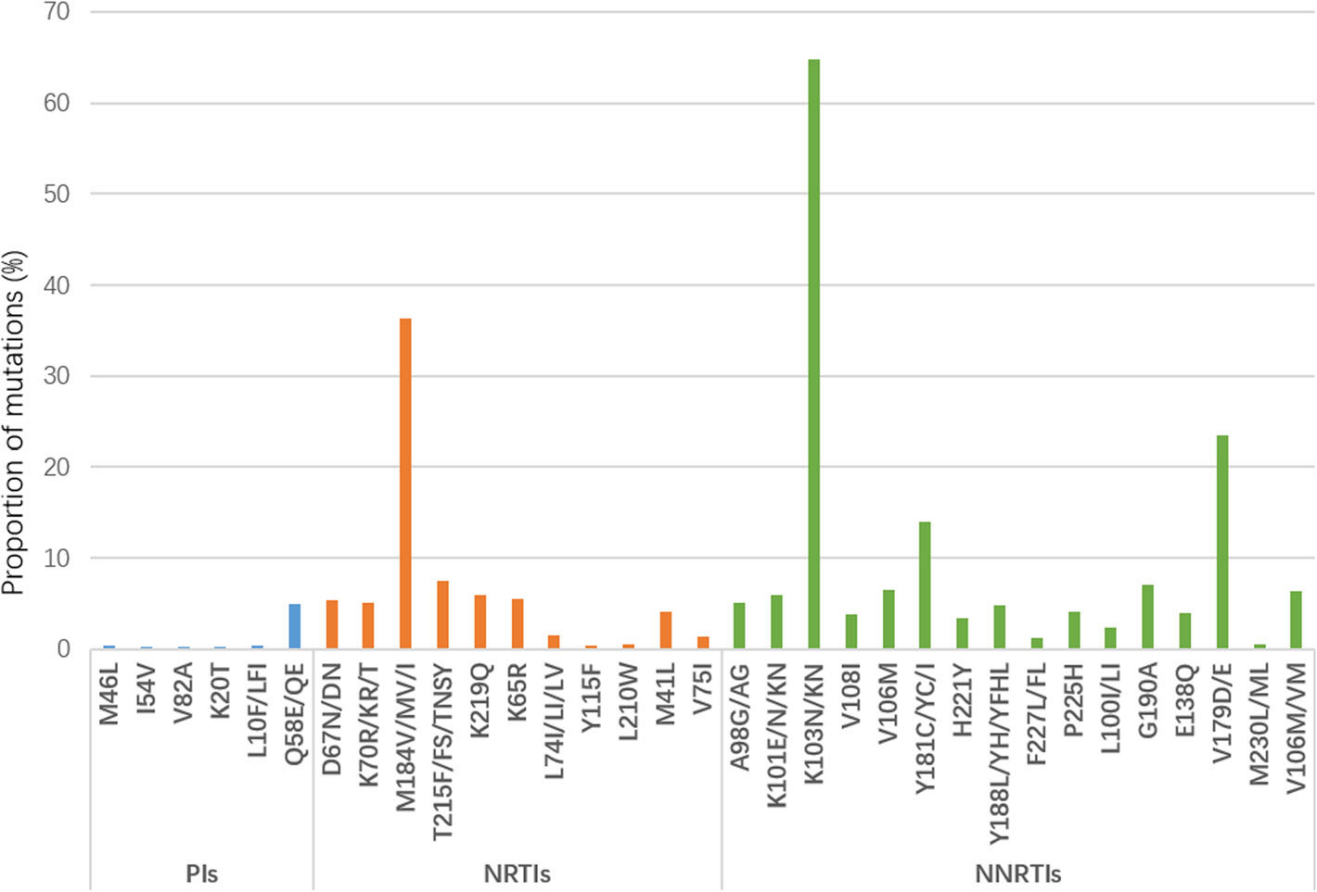
Drug resistance mutations to PIs, NRTIs and NNRTIs. Note: PIs mutations: M46I, I54V, V82A, K20T, L10F/LFI and Q58E/QE; NRTIs mutations: D67N/DN, K70R/KR/T, M184V/MV/I, T215F/FS/TNSY, K219Q, K65R, L74I/LI/LV, Y115F, L210W, M41L and V75I; NNRTIs mutation: A98G/AG, K101E/N/KN, K103N/KN, V108I, V106M, Y181C/YC/I, H221Y, Y188L/YH/YFHL, F227L/FL, P225H, L100I/LI, G190A, E138Q, V179D/E, M230L/ML and V106M/VM

The drug-resistant mutation frequency to NNRTIs (35.98%,567/1576) was much higher than that to NRTIs (14.72%,232/1576) and PIs (2.22%,35/1576) among PLWH with virological failure. The most common mutations in NNRTIs were K103N/KN (64.69%), V179D/E (23.47%) and Y181C/YC/I (14.00%), they were M184V/MV/I (36.29%), T215F/FS/TNSY (7.50%) and K219Q (5.92%) in NRTIs, and they were Q58E/QE (4.93%), L10F/LFI (0.39%) and M46L (0.39%) in PIs (Fig. 2).

## Discussion

The findings confirmed that the prevalence of virological failure on ART (41.81%) among PLWH was high in this area. A high prevalence of subtype CRF07_BC (95.62%) was observed among PLWH with virological failure on ART, which was different from other areas of China [24, 25]. Thus, it is a great challenge to successfully suppress HIV and decrease drug resistance in PLWH in this area.

Our study found that male PLWH and illiteracy were associated with a higher likelihood of incomplete viral suppression of virological failure on ART. Compared to females, males are more likely to discontinue from ART, which results in virological failure [26, 27]. Part of the reasons for this may be explained by (1) Compared to women, men generally have poorer healthcare-seeking behaviors, poorer ART uptake and a higher risk of immunotherapy failure observed in this study [28] (2) Women have a lower body mass index and are more likely to maintain a higher concentration of drugs in their bodies than men, so they are more likely to achieve viral suppression [29], (3) Men have more unhealthy behaviors such as smoking and drinking than women, which can lead to poorer adherence to medication and lower overall treatment success [30]. Illiteracy patients often lack sufficient understanding of the importance of ART and regular medication, as well as lack of communication skills with physicians [31, 32], which may increase the risk of virological failure. Therefore, it is necessary to develop effective measures to promote drug compliance of ART targeting males PLWH and illiteracy in Liangshan.

We also found that patients who had been on ART ≤12 months, and had changed ART regimens were more likely to experience virologic failure. Time on ART ≤12 months was associated with a high risk of virological failure than time on ART ≥12 months. This result was similar to a finding from a study in Zimbabwe, which showed that compare to time on ART ≥4 years, patients whose time on ART ≤4 years had more risk of treatment failure. Because the latter may have taken the wrong medicine or forgotten it due to lack of experience [30, 31]. Therefore, the current results all suggest the necessity of increasing the medication guidance, strengthening the management of treatment follow-up, and urging the patients to take medicine on time and following the prescribed amount [32]. Finally, the replacement of the ART regimen was also associated with virological failure. Patients who had replaced the ART regimen were more likely to experience treatment failure, possibly because they were not systematically tested for viral load and drug resistance before replacement [33]. However, our study didn’t obtain the information on the reasons for the replacement of the ART regimen. Therefore, the capacity of doctors should be improved, and the process of changing the ART regimen should be standardized. Viral load and drug resistance detection should be required before the replacement of the ART regimen.

Our studies found that the subtype CRF07_BC (95.62%) was most prevalent and was the predominant subtype, followed by CRF08_BC. CRF07_BC, which was one of the most prevalent CRFs in China [34–39] and was first identified in the intravenous drug use (IDU) population in Xinjiang in 1997 [38, 39]. CRF07_BC was first introduced into Yunnan and then spread into Sichuan and Gansu, and finally to Xinjiang along drug transporting routes [40, 41]. Liangshan is located along the drug trafficking routes from the “Golden Triangle” to the northwest and central China, and injection drug use was the main infection risk factors of HIV infection and about 33.2–56.9% of the new HIV-1 patients were drug users [42], and it can be assumed that CFR07_BC strain is prevalent in this area. Given the changing profile of the HIV epidemic with the shift of high-risk behaviors from IDU to sexual contact, the heterosexual transmission has surpassed injective drug use and became the predominant route of HIV transmission in this area [2, 3]. The dominant prevalent of CRF07_BC illustrated that infected IDUs are the main source of transmission to other populations. 

According to our findings, drug-resistant frequency to NNRTIs was much higher than that to NRTIs and PIs in this area among PLWH with virologic failure in ART. A similar pattern was also found in other places in China and low- and middle-income countries [43–46], but the prevalence of NNRTI resistance in this area is lower than in other places (50–90%) [47]. Thus, the high prevalence of virologic failure in ART and related low prevalence of drug resistance may be mainly attributed to poor treatment adherence, and strategies are needed to improve treatment adherence and reduce the treatment failure in ART.

There were several limitations in this study. First, this study was a cross-sectional survey, time-based sequence and cause-effect relationships among these variables cannot be established. Second, some HIV related behaviors were self-reporting, which may lead to recall bias. Third, some important factors of treatment failure in ART, such as ART adherence, should be considered as potential factors in our study, but such information did not collect during the follow-up. Fourth, the participants enrolled in the study were not newly diagnosed with HIV-1 infection, and some patients may have pretreatment drug resistance. Therefore, we could not identify the time of virologic failure and the resistance to ART before and after genotyping in the first virologic failure.

## Conclusions

The findings confirmed that the prevalence of incomplete viral suppression of ART virologic failure in ART among PLWH was high in this area. On the one hand, we should develop more suitable health education measures for medication compliance for this kind of population; on the other, the early identification of the drug resistance of PLWH with ART virologic failure are of great significance. Timely replacement of new regimens for PLWH with drug resistance could prevent additional drug resistance mutation or multiple drug resistance. Last but not the least, continuum in care and retention in care are important factors in preventing virological failure [48], and enhanced understandings of adherence and adherence interventions for less healthy individuals are required to reduce the virological failure. Also, understanding the genotype distribution and drug resistance may contribute to designing target preventive interventions for improving treatment efficiency, aiming for the selection of more effective therapeutic regimens to promote viral suppression and prevent HIV-1 transmission.

## Data Availability

The datasets used and analyzed during the current study are available from the corresponding author on reasonable request.

## Abbreviations

HIV: Human immunodeficiency virus
AIDS: Acquired Immune Deficiency Syndrome
ART: Antiretroviral therapy
PLWH: People living with HIV
VCT: Voluntary Counseling and Testing
NGO: Non-government organization
HSS: HIV sentinel surveillance
RT-PCR: Reverse transcription-polymerase chain reaction
OR: Odds ratios
CI: Confidence interval
NNRTI: Nonnucleoside reverse transcriptase inhibitor
NRTI: Nucleoside reverse transcriptase inhibitor
PI: Protease inhibitor
TDF: Tenofovir
3TC: Lamivudine
NVP: Nevirapine
EFV: Efavirenz
AZT: Zidovudine
LPV/r: Tipranavir/ritonavir
ATV/r: Atazanavir/ritonavir
DRv/r: Darunavir/ritonavir
FPV/r: Tipranavir/ritonavir
IDV/r: Indinavir/Ritonavir
NFV: Nelfinavir
SQV/r: Ritonavir-boosted saquinavir
TPV/r: Tipranavir/ritonavir
ABC: Abacavir
D4T: Stavudine
DDI: Didanosine
FTC: Emtricitabine
ETR: Etravirine
RPV: Rilpivirine
